# Treatment of Cholera

**Published:** 1850-11

**Authors:** B. F. Mullen

**Affiliations:** Madison, Ind.


### ARTICLE III.

*Treatment of Cholera.*—By B. F. Mullen, M. D., of Madison, Ind.

Prof. Evans—Dear Sir: Having observed through the medium of the press notices of the appearance of Cholera in your city, I take the liberty of presenting for your consideration, the treatment employed by me in that disease. I may also state that the same treatment was adopted by my brother, Dr. A. J. Mullen, of Napoleon, and, singularly enough, too, simultaneously with myself.

To the treatment: If it was ascertained that the patient had lately partaken of indigestible or irritating food, an emetic composed of mustard and salt—of each a table-spoon full—was given, dissolved in warm water. This was followed by the following:

℞.——Hyd. chlo. mit., grs. xxx.
Pulv. Camphor, grs. xii.
" Capsicum, grs. vi.
" Acet. Plumbi, grs. xviii.
M. ft. chart. no. vj.

These powders are given as frequently as every 15 minutes, if the rice-water discharges are profuse and frequent, and less frequently if the case is a mild one. Is severe vomiting present? a large sinapism is laid on the bowels: in collapse, or cases bordering upon that state, the patient should be surrounded with *corn ears*, previously heated in boiling water, and wrapped in flannel cloths or blankets,—the bed removed to the middle of the room in order to admit a free current of atmosphere, no rubbing or annoying the patient, who is all the time to be kept in a horizontal position, and perfectly quiet.

As the rice-water discharges cease, the first ingredient in the powder is increased and the others diminished.

Iced Slippery Elm water is allowed the patient freely; for the cramps nothing, in my opinion, can be done, for they are occasioned by the absence of serum in the circulating system; for, how often do we meet with cramps in persons who have suffered from profuse Hemorrhage.

I thus inform you of the treatment employed, as it has been most successful. My brother, Dr. A. J. Mullen, has lost only six patients out of some two hundred, and I can find but ten fatal cases in all my Cholera practice, both in this city and at Napoleon, where I was called during the prevalence of that disease last summer. The treatment of the consecutive fever must be founded upon general principles. Death, when it occurs, is evidently from effusion in the ventricles.

I need scarcely say to you that the diarrhœa preceding an attack of Cholera is no ways identified with the diarrhœa usual to our country or season. Whilst in the hospitals in Mexico, (where I had the honor to serve,) I had the opportunity of observing diarrhœa in all its phases. Of course I had no difficulty in diagnosticating between Serous Diarrhœa and the other forms.

